# Electroacupuncture modulates glutamate neurotransmission to alleviate PTSD-like behaviors in a PTSD animal model

**DOI:** 10.1038/s41398-023-02663-4

**Published:** 2023-11-22

**Authors:** Mudan Cai, Hee Ra Park, Eun Jin Yang

**Affiliations:** KM Science Research Division, Korea Institute of Oriental Medicine (KIOM), 1672 Yuseong-daero, Yuseong-gu, Daejeon, 34054 Korea

**Keywords:** Hippocampus, Depression

## Abstract

Post-traumatic stress disorder (PTSD) is a mental disorder that develops after exposure to a traumatic event. Owing to the relatively low rates of response and remission with selective serotonin reuptake inhibitors as the primary treatment for PTSD, there is a recognized need for alternative strategies to effectively address the symptoms of PTSD. Dysregulation of glutamatergic neurotransmission plays a critical role in various disorders, including anxiety, depression, PTSD, and Alzheimer’s disease. Therefore, the regulation of glutamate levels holds great promise as a therapeutic target for the treatment of mental disorders. Electroacupuncture (EA) has become increasingly popular as a complementary and alternative medicine approach. It maintains the homeostasis of central nervous system (CNS) function and alleviates symptoms associated with anxiety, depression, and insomnia. This study investigated the effects of EA at the GV29 (Yintang) acupoint three times per week for 2 weeks in an animal model of PTSD. PTSD was induced using single prolonged stress/shock (SPSS) in mice, that is, SPS with additional foot shock stimulation. EA treatment significantly reduced PTSD-like behavior and effectively regulated serum corticosterone and serotonin levels in the PTSD model. Additionally, EA treatment decreased glutamate levels and glutamate neurotransmission-related proteins (pNR1 and NR2B) in the hippocampus of a PTSD model. In addition, neuronal activity and the number of Golgi-impregnated dendritic spines were significantly lower in the EA treatment group than in the SPSS group. Notably, EA treatment effectively reduced glutamate-induced excitotoxicity (caspase-3, Bax, and pJNK). These findings suggest that EA treatment at the GV29 acupoint holds promise as a potential therapeutic approach for PTSD, possibly through the regulation of NR2B receptor-mediated glutamate neurotransmission to reduce PTSD-like behaviors.

## Introduction

Post-traumatic stress disorder (PTSD) is a psychiatric disorder that develops after a traumatic event, such as sexual assault, warfare, or traffic collisions. In the United States, approximately 3.5% of adults experience PTSD within 1 year, while ~9% develop PTSD at some stage during their lifetime [1]. In preclinical studies, single prolonged stress (SPS) is commonly used to induce PTSD in animal models [2], with such models exhibiting phenotypes similar to those of patients with PTSD, including impaired extinction retention, hyperarousal, cognitive dysfunction, sleep disruption, and anxiety [3]. The neural and molecular mechanisms underlying PTSD involve the dysregulation of various processes, including the hypothalamus–pituitary–adrenocortical (HPA) axis, neurotransmitters, and cellular adaptations (signal transduction and apoptosis) [3]. Among these dysregulations, an important aspect of the complex pathophysiology of PTSD is the alteration of glutamate neurotransmitters, which depends on whether neurotransmission in the brain regions is disrupted. In addition, neurotransmitter imbalances, such as excessive production of excitatory neurotransmitters, can contribute to excitotoxicity and lead to cell damage. This imbalance in neurotransmitters is believed to underlie the many behavioral changes observed in animals and patients with PTSD. Particularly, the N-methyl-d-aspartate (NMDA) receptor subunit NR2B has been implicated in modulating functions such as learning, memory processing, and neuronal pattern formation [4]. Furthermore, Zhang et al. demonstrated that glutamate-induced cell damage is associated with the regulation of genes, such as B-cell lymphoma 2 (Bcl-2), BCL-2-associated X protein (Bax), and caspase-3 and mitochondrial cytochrome c [5, 6].

Anxiolytics and antidepressants are currently used as the primary treatment modalities for PTSD. Notably, selective serotonin reuptake inhibitors (SSRIs) such as sertraline and paroxetine have been approved by the Food and Drug Authority for PTSD treatment. Regrettably, despite undergoing sufficient trials, the rates of response and remission for PTSD treatment are ~60% and 30%, respectively [7, 8]. Another crucial limitation is the importance of avoiding abrupt discontinuation of treatment with SSRIs. Consequently, despite the availability of these treatments, effective strategies for patients with PTSD are still lacking. Therefore, exploring and developing effective treatment strategies for PTSD is imperative.

Electroacupuncture (EA) is a widely used form of complementary and alternative medicine (CAM) that combines traditional acupuncture techniques with electrical stimulation and offers a distinct therapeutic approach. Both preclinical and clinical studies have identified several acupoints, such as GV20 (Baihui), BL23 (Shenshu), HT7 (Shenmen), and PC6 (Neiguan), that have demonstrated significant benefits in the treatment of PTSD [9, 10]. These specific acupoints have a significant impact on alleviating symptoms, such as reducing anxiety, depression, and fear responses; improving sleep patterns; and mitigating spatial learning and memory deficits. These beneficial effects are achieved through the regulation of various pathways, including the HPA axis, brain-derived neurotrophic factor (BDNF)-tropomyosin receptor kinase B, Kelch-like ECH-associated protein 1-nuclear factor erythroid 2-related factor 2, and mammalian target of rapamycin pathways [9]. In addition, Jiang et al. demonstrated that combining acupuncture treatment at the GV20 and GV29 (Yintang) acupoints alleviated the anxiety index through an elevated plus maze test, reversed pathological injury, and reduced microglia in the hippocampus of a PTSD model [11]. Moreover, EA stimulus at the GV29 and GV20 acupoints reduced depression-like behaviors in chronic unpredictable mild stress and maternal separation rats [12, 13]. However, the specific effect of EA treatment at the GV29 acupoint in an animal model of PTSD induced by a single prolonged stress/shock (SPSS) and the underlying mechanisms are yet to be understood. Hence, this study aimed to investigate the effects of EA at the GV29 acupoint three times per week for 2 weeks in an animal model of PTSD.

## Materials and methods

### Animals

Adult male C57BL/6N mice (21–23 g, 8 weeks old) were purchased from Dae Han Biolink (Eumseong-gun, Chungcheonbuk-do, Korea). Five mice were housed under specific pathogen-free control conditions, and water and food were provided ad libitum. Mice were habituated for a week in a constant temperature (21 ± 3 °C) and humidity (50 ± 10%) with a 12 h light/dark cycle. The mice were cared for and treated in accordance with the National Institute of Health Guide and the animal care guidelines of the Korea Institute of Oriental Medicine (KIOM). The mice were randomly grouped into three groups: a control group (CON, n = 22), an enhanced SPSS group (SPSS, n = 22), and a SPSS + GV29 group (n = 22). The experimental protocol was approved by the Institutional Animal Care Committee of KIOM (protocol number: 21–104).

### Enhanced single prolonged stress/shock animal model

The SPS model of PTSD was established as described previously [14]. Briefly, the mice were first immobilized for 4 h on restraint tubes and then immediately subjected to forced swimming for 20 min in 23–24 °C water. Next, animals were allowed to rest for 15 min in the cage and then exposed to isoflurane until loss of consciousness [2]. Finally, two foot shocks (1 mA, 4 s) were delivered through the grid floor in the chamber. On the seventh day, the mice were subjected to a fifth foot shock (1 mA, 4 s) in the enhanced SPS animal model.

### Electroacupuncture treatment and experimental design

**Fig. 1:**
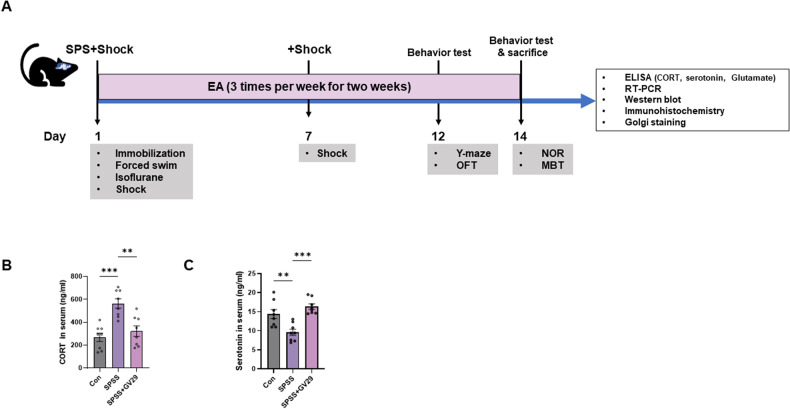
Generation of SPSS-induced PTSD animal model. **A** Experimental timeline. 8 weeks old mice are allowed to habituate 1 week prior to inducing PTSD. EA treatment was conducted at GV29 3 times per week for 2 weeks. Under the experimental timeline, two sets of experiments were conducted. The first set consisted of the Y-maze and NOR tests and second set included OFT and MBT. **B** Quantitative analysis of the levels of serum CORT in the SPSS-induced PTSD animal model (n = 8/group). **C** Quantitative analysis of the levels of serum serotonin in the PTSD model (n = 8/group). Each open circle in the graphs represents each mouse. Data are represented as the means ± SEM (*p < 0.05). SPS single prolonged stress, EA electroacupuncture, OFT open field test, NOR novel object recognition test, MBT marble burying test, CORT corticosterone.

EA treatment was performed as previously described [15]. Briefly, the mice were subjected to EA treatment at the Governor Vessel 29 acupoint (GV29). Visually, the GV29 acupoint was located midline between the brows [16, 17]. After anesthetization with isoflurane (Hana Pharm Co. Ltd., Hwaseong, Korea), the anode and cathode of the electrical stimulator (Partner-1; Daejeon, South Korea) were connected to the acupuncture needles, and electrical stimulation pulses were applied for 15 min (1 mA, 2 Hz) at the acupoint. EA treatment was performed three times per week for 2 weeks. The experimental scheme included EA treatment, behavioral tests, and brain tissue collection (Fig. 1A).

### Behavioral tests

#### Y-maze test

The Y-maze test was conducted on the next day following the fifth EA treatment, as previously described [15]. The mice were placed in a Y-maze apparatus with three black arms positioned at 120° angles between the arms. The sequence of arms entered was recorded for 8 min. A valid alternation was defined as entry into three different arms in a pattern (i.e., ABC, BCA, or CAB, but not CAC or ACA). Spontaneous alternation was calculated using the following formula: spontaneous alternation (%) = (number of alternations)/(total arm entries − 2) × 100. The total number of arm entries was used as an indicator of locomotor activity.

#### Novel object recognition test

On the 13th day before the sixth EA treatment, the mice were performed novel object recognition test (NOR). Mice were acclimated to the open field apparatus (30 × 30 × 30 cm) without any objects for 10 min. At 1 h after the acclimation period, the mice were returned to the same box containing two identical familiar objects for 10 min. The next day after completion of the last EA treatment, the animals were reintroduced to the same box and allowed to freely explore for 7 min while being recorded by a video camera. The box still contained two objects, but one of the familiar objects was replaced with a novel object that had a different shape but with a similar texture. To assess the object preference, the object preference ratio was calculated using the following equation: object preference ratio (%) = (*T*_novel_)/(*T*_novel_ + *T*_familiar_) × 100. During the probe trial of this behavioral test, it was expected that the total exploration time (T_novel_ + T_familiar_) would exceed 10 s. If the total exploration time was less than 10 s, the mouse was excluded from the analysis.

#### Open field test

The open field test (OFT) is typically used to assess emotion and locomotor activity. For the second set of animals, the OFT was conducted on the next day after the fifth EA treatment. The mice were individually placed in an open field apparatus with black walls and a floor (30 × 30 × 30 cm) and allowed to freely explore the environment for 20 min. Mouse movements were recorded using a video-based Ethovision XT System (Noldus Information Technology BV). The locomotor activity of mice was assessed by measuring the total distance and duration of movement within the box. The exploratory activity was evaluated by counting the total number of line crossings [18]. To assess anxiety-like behavior, the time spent in the central zone of the open field apparatus was measured as the anxiety index.

#### Marble-burying test

For the second set of animals, the marble-burying test (MBT) was conducted on the day following completion of the sixth EA treatment. Twenty glass marbles were placed equidistantly in a 5 × 4 pattern within the testing cage. The mice were allowed to freely explore the cage with marbles for 20 min. The anxiety index was evaluated based on the number of marbles buried by mice. A marble was considered to be buried when at least two-thirds of its size was covered with a burying substrate [19].

### Tissue preparation

After completing the behavioral test, the mice were anesthetized using avertin (Sigma, St. Louis, MO, USA), and blood was collected from the heart. In preparation for the western blotting analysis, hippocampus regions were dissected and promptly stored at −70 °C. For immunohistochemistry, the mice underwent transcardial perfusion with phosphate-buffered saline (PBS) to remove blood. Subsequently, the brains were carefully removed and post-fixed overnight at 4 °C in 4% paraformaldehyde. The following day, brains were transferred to a solution of 30% sucrose in PBS and stored at 4 °C until they were ready for sectioning. The coronal plane sections of the frozen brains were obtained using a cryostat (Leica Microsystems AG, Germany) at a thickness of 30 μm. After sectioning, the slices were carefully placed in a storage solution at 4 °C to ensure their preservation until further analysis.

### Corticosterone, serotonin, and glutamate assays

Blood samples were centrifuged at 3000 rpm at 4 °C for 10 min, and the supernatant was transferred into the new tube. The obtained supernatants were centrifuged again at 12,000 rpm at 4 °C for 10 min, and the resulting supernatant, referred to as serum, was once again transferred into the new tube. Finally, serum samples were analyzed using a corticosterone (CORT) assay kit (EIACORT, Thermo Fisher Scientific, Waltham, MA, USA) and a serotonin ELISA kit (MBS1601042, Mybiosource, San Diego, CA, USA), according to the manufacturer’s instructions, to determine the corticosterone and serotonin levels.

General glutamate assay kits (ab83389, Abcam, Cambridge, MA, USA) were used to assess the hippocampal glutamate levels. To ensure accurate measurements, sample concentrations were optimized to ensure that they fell within the range of the standard curve, and the levels of glutamate in the hippocampal tissues were detected using the respective assay kits. The optical density (OD) value of each well was measured using a plate reader (Molecular Devices, San Jose, CA, USA) at an absorption wavelength of 450 nm for each assay.

### Western blots

Western blotting was performed as described previously [15]. The hippocampal tissue was homogenized in a radioimmunoprecipitation assay buffer (Biosesang, Gyeonggi-do, Korea) supplemented with phosphatase and protease inhibitors (Thermo Fisher Scientific). The loaded proteins (20 μg) were separated on Bolt 4–12% Bis-Tris Plus gels (Thermo Fisher Scientific) and subsequently transferred onto polyvinylidene difluoride membranes (Bio-Rad, Hercules, CA, USA). The transferred membranes were then incubated with primary antibodies overnight at 4 °C, followed by washing with Tris-buffered saline Tween. Next, the membranes were incubated with the corresponding secondary antibodies (Santa Cruz Biotechnology, Santa Cruz, CA, USA) for 2 h at room temperature. After washing the membranes, the immunoblots were visualized using a ChemiDoc Imaging System (Bio-Rad) and quantified using ImageJ software (version 1.46j; National Institutes of Health, Bethesda, MD, USA). The primary antibodies used were as follows: NMDA receptor 1 (Ser896) (pNR1 (ser 896)) (1:500, PA5–37589; Thermo Fisher Scientific), NMDAR subunit 1 (NR1) (1:1000, PA3-102; Thermo Fisher Scientific), NMDAR subunit 2B (NR2B) (1:1000, ab65783; Abcam), tubulin (1:1000, ab4074; Abcam), Bax (1:1000, sc-7480; Santa Cruz Biotechnology, Santa Cruz, CA, USA), Bcl-2 (1:1000, sc-7382; Santa Cruz Biotechnology), phospho-SAPK/JNK (Tyr183/Tyr185) (pJNK) (1:1000, 9251 s; Cell Signaling Technology, Danvers, MA, USA), and c-Jun N-terminal kinases (JNKs) (1:1000, 9252 s; Cell Signaling Technology).

### Immunohistochemistry

For immunohistochemical staining [20], the sections were incubated in 3% H_2_O_2_ to block endogenous peroxidase activity. Thereafter, the sections were placed in a blocking solution for 1 h and incubated overnight at 4 °C with the primary antibodies (NR2B (1:1000, ab65783; Abcam)) and glutamate decarboxylase 67 (GAD67) (1:1000, ab213508; Abcam). The following day, the sections were incubated with the appropriate secondary antibody for 2 h. Subsequently, for visualization, the sections were incubated in an Avidin-Biotin Complex solution (Vector Laboratories, Burlingame, CA, USA) for 1.5 h, followed by incubation in the 3,3′-diaminobenzidine peroxidase substrate solution (Vector Laboratories) for approximately 5 min. To analyze NR2B staining, the hippocampus was further divided into subregions, including the CA1, CA3, and dentate gyrus (DG), and the staining density was determined in each of these regions. For GAD67 staining, the number of GAD67-positive cells was counted blindly following a rigorous protocol to ensure an unbiased analysis.

### Real-time quantitative polymerase chain reaction (RT-qPCR)

Real-time quantitative polymerase chain reaction (RT-qPCR) was performed as previously described [20]. RNA was extracted from the hippocampal tissues using a total RNA extraction kit (17221; Intron Biotechnology, Seongnam-Si, Korea) according to the manufacturer’s protocol. cDNA was synthesized using a cDNA synthesis kit (1708891; Bio-Rad) according to the manufacturer’s instructions. After cDNA synthesis, each sample template was combined with SYBR Supermix (1725121; Bio-Rad), gene-specific primers, and nuclease-free H_2_O. The RT-PCR was performed using the following cycle parameters: an initial denaturation step at 95 °C for 30 s, followed by 40 cycles of denaturation at 95 °C for 15 s, and annealing/extension at 60 °C for 1 min. The gene-specific primers used in this study were synthesized by Bioneer Corp. (Daejeon, Republic of Korea). The following primers were used: activity-regulated cytoskeletal-associated protein (Arg 3.1) (Accession No: NM_018790); early growth response 1(Egr-1) (Accession No: NM_007913); BDNF (Accession No: NM_007540); vesicular GABA transporter (vGAT) (Accession No: NM_009508); vesicular glutamate transporter 1 (vGLUT1) (Accession No: NM_182993); vGLUT2 (Accession No: NM_080853); caspase-3 (Accession No: NM_009810); BAX (Accession No: NM_007527); Bcl-2 (Accession No: NM_009741); and actin (Accession No: NM_007393). Actin was used as the housekeeping gene.

### Golgi staining

Golgi staining was performed using the FD Rapid Golgistain kit (PK401; FD NeuroTechnology, Columbia, MD, USA). The mice were sacrificed, and their brains were quickly removed and immediately placed in the impregnation solution provided in the kit. Golgi staining was performed according to the manufacturer’s instructions. Thereafter, brain samples were coronally sectioned into 100-µm-thick using a cryostat. For quantification, the hippocampal region was subdivided into the CA1 and DG regions. The number of dendritic spines was counted across five sections from each mouse, with a diameter of 20 micrometers.

### Statistical analysis

The sample size for the study was not determined using any predetermined statistical method. To quantify staining in the hippocampal-specific regions, slice images were captured using a microscope (Olympus BX53, Tokyo, Japan). Cells were counted from bilateral brain sections per animal by a person blinded to the sample grouping. Data were expressed as the mean ± standard error of the mean (SEM). Statistical analyses were performed using Prism software (version 9.0; GraphPad, La Jolla, CA, USA). If normal distribution was confirmed using the Kolmogorov–Smirnov test, we conducted statistical analysis using the one or two-way analysis of variance, followed by Tukey’s test for conducting multiple comparisons. Statistical analysis was performed using the Kruskal-Wallis test for non-normally distributed data and followed by Dunn’s multiple comparison test. If the sample size was small, we used Shapiro-Wilk test to test for normal distribution. Statistical significance levels were established at *p < 0.05, **p < 0.01, and ***p < 0.001.

## Results

### EA attenuates cognitive impairments and anxiety

**Fig. 2:**
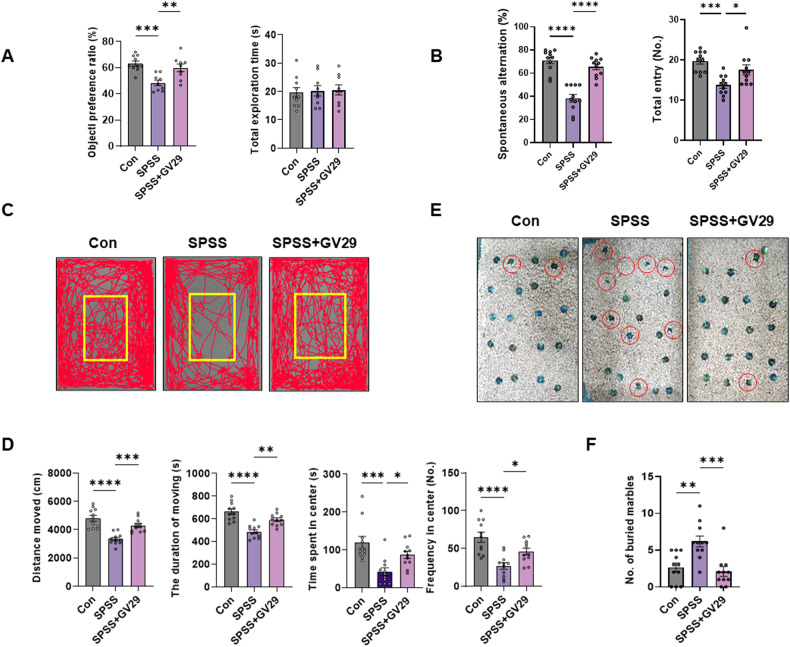
EA attenuates PTSD-like behaviors in the PTSD animal model. **A** Data showing the results of the NOR test (Con, n = 10; SPSS, n = 9; SPSS + GV29, n = 9). The graphs showing quantitative object preference ratio and total exploration time. **B** Data showing the results of the Y-maze test. The graphs show quantitative spontaneous alteration and total entry (n = 11/group). **C** Representative track plot of distance moved in the open field box. **D** Data show the results of OFT. The graphs show the total distance moved, the duration of moving, the time spent in center, and the frequency to center zone (n = 11/group). **E** Representative images after free exploration post-MBT. **F** Data showing the MBT. The graph shows the number of buried marbles (n = 11/group). Each open circle in the graphs represents each mouse. Data are presented as the means ± SEM (*p < 0.05, **p < 0.01, ***p < 0.001, and ****p < 0.0001). CON control, SPSS SPS + shock.

Serum CORT levels were significantly higher in the SPSS group than in the control group. However, EA treatment resulted in a remarkable decrease in serum CORT levels compared to those in the SPSS group (Fig.1B). Moreover, the serum serotonin levels were regulated by EA treatment in the SPSS model group (Fig.1C). The animal model exhibited phenotypes similar to those of patients with PTSD. Particularly, the SPS-induced PTSD animal model showed molecular alterations similar to those in patients with PTSD, such as serum levels of CORT and serotonin, and these alterations may induce prolonged behaviors. In the NOR test, the SPSS + GV29 group showed significantly longer exploration time of novel objects than the SPSS group, indicating an improvement in cognitive function, particularly spatial and working memory (Fig. 2A). In the Y-maze test, SPSS induction led to a significant decrease in spontaneous alternations, indicating an impairment of hippocampus-dependent spatial memory. However, EA treatment effectively restored spontaneous alternations compared to the SPSS animals. Furthermore, the total entries, which serve as indicators of locomotor activity, were also attenuated by EA treatment compared to the SPSS animals (Fig.2B). For anxiety levels assessed using the OFT and MBT, the SPSS group exhibited a significant decrease in locomotor activity, including the total distance moved and duration of movement in the open field box. Meanwhile, the SPSS + GV29 group showed an increase in these parameters (Fig. 2C). Moreover, anxiety-related parameters, such as time spent in the center and frequency to the center zone, were attenuated by EA treatment compared to SPSS animals (Fig. 2D). Additionally, in the MBT, the number of buried marbles was lower in the SPSS + GV29 group than in the SPSS group (Fig. 2E, F). Collectively, these behavioral test findings indicate that EA treatment effectively attenuated impairments in working memory and spatial memory and reduced anxiety levels.

### EA modulates glutamate transmission

**Fig. 3:**
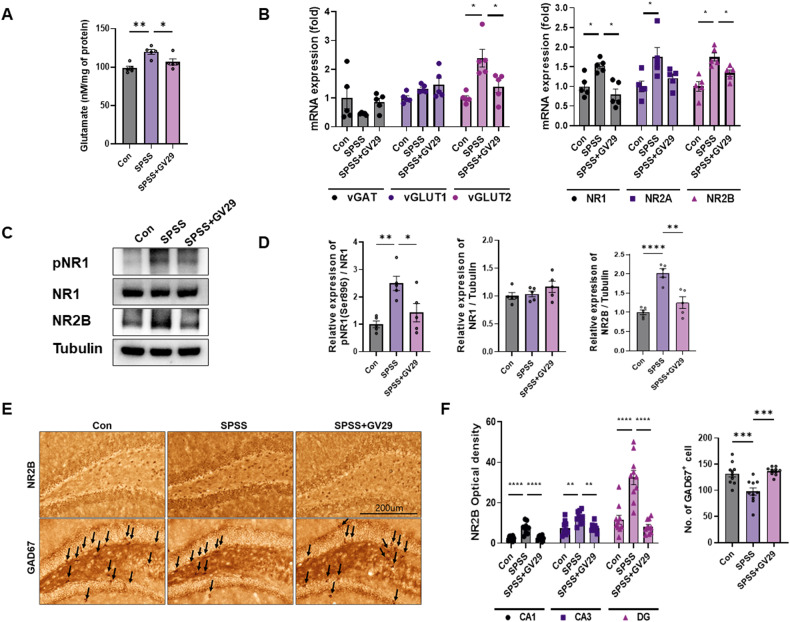
EA modulates glutamate transmission in the SPSS-induced PTSD animal model. **A** The graphs show the glutamate levels in each group (n = 5/group). **B** Quantitative analysis of the relative mRNA expression of vGAT, vGluT1, vGluT2, NR1 NR2A, and NR2B in the hippocampus of each group (n = 5/group). **C** Representative western blot images depicting alterations in glutamate transmission-related proteins, namely, pNR1(ser896), NR1, and NR2B, in the hippocampus of mice from each group. Tubulin was used as the loading control. **D** Quantitative analysis of the bands showing the levels of pNR1(ser896)/NR1, NR1/Tubulin, and NR2B/Tubulin in each group (n = 5/group). **E** Representative images showing NR2B and GAD67 immunostaining in the DG of the hippocampus in each group. **F** Quantification analysis of the optical density of NR2B in CA1, CA3, and DG regions of the hippocampus and GAD67-positive cells in the hippocampus in each group (n = 5/group). Scale bar, 200 μm. Each open circle in the graphs represents each mouse. Data are presented as the means ± SEM (*p < 0.05, **p < 0.01, ***p < 0.001, and ****p < 0.0001). CON control, SPSS SPS + shock. vGAT vesicular GABA transporter vGLUT1 vesicular glutamate transporter 1, vGLUT2 vesicular glutamate transporter 2, NR1 NMDA receptor subunits NR1, NR2A NMDA receptor subunits NR2A, NR2B NMDA receptor subunits NR2B, GAD67 glutamate decarboxylase 67.

Glutamate dysregulation and glutamate neurotransmission dysfunction are increasingly recognized features of PTSD [8]. Regarding the effect of EA on glutamate transmission, glutamate in the hippocampus were significantly increased in the SPSS group compared with control. Meanwhile, glutamate levels were lower in the SPSS + GV29 group than in the SPSS group, indicating the regulation of glutamate transmission by EA treatment (Fig. 3A). In addition, our results revealed a significant increase in the glutamate transmission-related mRNA levels of vGluT2, NR1, NR2A, and NR2B in the SPSS group than in the control group. However, these levels were lower in the SPSS + GV29 group than in the SPSS group (Fig. 3B). The mRNA levels of vGAT were 54% lower in the control group than in the SPSS group (p > 0.05), while they were higher in the SPSS + GV29 group than in the SPSS group. Additionally, the levels of pNR1(ser896)/NR1 and NR2B/Tubulin in the hippocampus were significantly higher in the SPSS group than in the control group, whereas they were lower in the SPSS + GV29 group (Fig. 3C, D). Further, the OD values of NR2B in the CA1, CA3, and DG regions of the hippocampus were significantly higher in the SPSS group than in the control group, whereas they were significantly lower in the SPSS + GV29 group (Fig. 3E, F). In addition, the number of GAD67-positive cells in the hippocampus was significantly higher in the SPSS + GV29 group than in the SPSS group (Fig. 3E, F). These data show that EA treatment effectively attenuates glutamate neurotransmission by regulating the NR2B receptor.

### EA modulates the number of dendritic spines

**Fig. 4:**
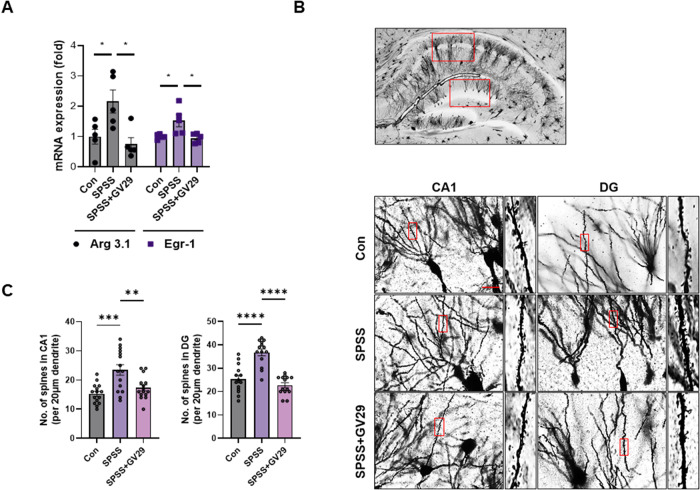
EA-modulated dendritic spines in the SPSS-induced PTSD animal model. **A** Quantitative analysis of the relative mRNA expression of Arg 3.1 and Egr-1 in the hippocampus of each group (n = 5/group). **B** Representative images showing Golgi staining in the hippocampus in each group. **C** Quantification of the number of dendritic spines in CA1 and DG regions of the hippocampus (n = 5/group). Scale bar, 20 μm. Each open circle in the graphs represents each mouse. Data are presented as the means ± SEM (*p < 0.05, **p < 0.01, ***p < 0.001, and ****p < 0.0001). CON control, SPSS SPS + shock, CA1 Cornu Ammonis 1, DG dentate gyrus.

The mRNA levels of Arg 3.1 and Egr-1 were significantly higher in the SPSS group than in the control group, whereas it was lower in the SPSS + GV29 group than in the SPSS group (Fig. 4A). Next, Golgi staining showed a significantly higher number of dendritic spines within the CA1 and DG regions of the hippocampus in the SPSS group than in the control group, whereas the number was lower in the SPSS + GV29 group than in the SPSS group (Fig. 4B–C). These results demonstrate that EA treatment regulates excitatory neuronal activity in PTSD.

### EA regulates glutamate excitotoxicity

**Fig. 5:**
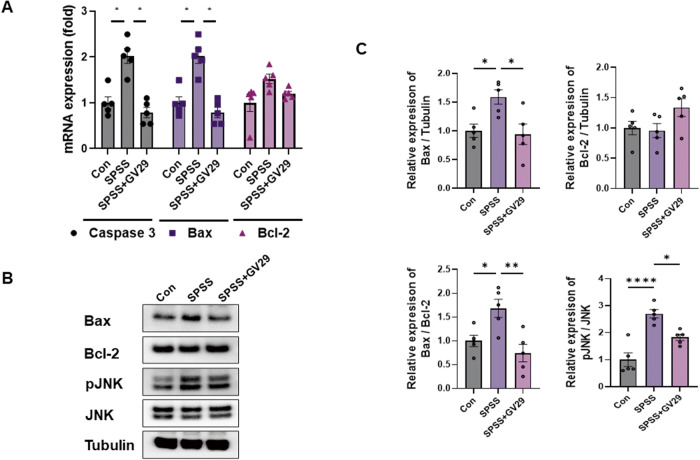
EA-regulated glutamate excitotoxicity in SPSS-induced PTSD animal model. **A** Quantitative analysis of the relative mRNA expression of caspase-3, Bax, and Bcl-2 in the hippocampus of each group (n = 5/group). **B** Representative western blot images depicting alterations in glutamate excitotoxicity-related proteins, including Bax, Bcl-2, and pJNK, in the hippocampus of mice from each group. Tubulin was used as the loading control. **C** Quantitative analysis of the bands showing the levels of Bax/Tubulin, Bcl-2/Tubulin, Bax/Bcl-2, and pJNK/JNK in each group (n = 5/group). Tubulin was used as the loading control. Each open circle in the graphs represents each mouse. Data are presented as the means ± SEM (*p < 0.05, **p < 0.01, ***p < 0.001, and ****p < 0.0001). CON: control, SPSS SPS + shock.

Glutamate excitotoxicity refers to the excessive activation of glutamate receptors leading to neuronal damage. Glutamate excitotoxicity may be related to hippocampal neuron degeneration and memory-related (re-experiencing) symptoms in patients with PTSD [21]. Therefore, we investigated the intracellular molecular mechanisms underlying the effects of EA treatment in a PTSD model. The results showed that the mRNA levels of glutamate excitotoxicity-related markers, including caspase-3, Bax, and Bcl-2, in the hippocampus were significantly higher in the SPSS group than in the control group. However, these levels were lower in the SPSS + GV29 group than in the SPSS group (Fig. 5A). Furthermore, the protein expression of the excitotoxicity-related markers Bax, Bax/Bcl-2 ratio, and pJNK in the hippocampus was significantly higher in the SPSS group than in the control group, whereas it was lower in the SPSS + GV29 group than in the SPSS group (Fig. 5B, C). Collectively, these results suggest that EA may have a modulatory effect on glutamate excitotoxicity in SPSS-induced PTSD.

## Discussion

PTSD is a mental disorder that develops after a traumatic event. The efficacy of EA treatment for PTSD has been reported in both human and animal studies [9, 10]. However, the precise mechanism underlying the therapeutic effects of EA remains unclear. This study elucidated the molecular mechanisms of EA treatment by establishing a correlation between PTSD-like behaviors and the balance of neuronal transmission in the hippocampus of mice with SPSS-induced PTSD. We demonstrated the effects of EA treatment at the GV29 acupoint in an animal model of PTSD. In the enhanced SPSS-induced PTSD model, additional foot shocks were provided after SPS to better mimic the clinical characteristics of PTSD (e.g., anxiety, cognitive impairment, and failure of fear extinction), which PTSD patients show that re-experiencing traumatic events and avoid stimuli [14, 22–24]. This model sufficiently reflected PTSD symptoms and satisfied face validity [25]. Many studies have shown that CORT levels in the serum are increased in patients with PTSD, and increased CORT levels can affect anxiety-like symptoms and are related to the progression or exacerbation of PTSD [26]. Moreover, serotonin is also a treatment target for PTSD, anxiety, and depression as it is significantly decreased in the serum [27]. Our results showed that EA treatment effectively modulated the levels of CORT and serotonin. The increased CORT levels indicate that the SPSS-induced PTSD animal model was subjected to extremely stressful situations. Alterations in CORT and serotonin levels follow anxiety and fear memory impairments in PTSD [28]. Patients with PTSD also exhibit impaired object and social recognition, which may underlie the avoidance and symptoms of negative cognition, such as social estrangement or diminished interest in activities [29]. In preclinical studies, SPS animal models also showed cognitive impairment in the NOR and Y-maze tests [14, 30, 31], which indicate hippocampal damage. Our results indicated that object preference for the novel object, as an index of recognition memory, was increased by EA treatment. Furthermore, spontaneous alternation, as an indicator of spatial memory in the Y-maze test, was significantly higher in the SPSS + GV29 group than in the SPSS group. In addition, the total entries, which indicated locomotor activity, were increased by EA treatment. The relationship between anxiety and cognitive ability is bidirectional. Anxiety can disrupt cognitive abilities, leading to difficulties in concentration, memory, and problem-solving. In addition, cognitive impairment can increase anxiety, as individuals may become more anxious about their diminished cognitive performance [32]. Therefore, the association between anxiety and cognitive ability is complex. In the current study, anxiety-related indices, such as locomotor activity, duration in the center, frequency in the center, and number of buried marbles, in the OFT and MBT improved in EA treatment. These data suggest that EA treatment attenuates PTSD-like behaviors associated with hippocampus-dependent impairments.

Accumulating evidence shows abnormal glutamatergic function in mood, anxiety, and trauma-related disorders, and impaired glutamate neurotransmission is now widely recognized to play a key role in the pathophysiology of stress-related psychiatric conditions, including PTSD [8]. Nishi et al. suggested that the glutamatergic system not only plays a vital role in the stress response and PTSD, but is also associated with PTSD diagnosis or severity [33]. Therefore, we first measured the effects of EA on synaptic transmission-related markers in the hippocampus of the animal model and subsequently confirmed the level of glutamate in the hippocampus. The results showed that EA treatment decreased the level of glutamate in the hippocampus in the SPSS group. Glutamate synthase markers (mRNA levels of vGluT2) and synaptic transmission markers (mRNA levels of NR1, NR2A, and NR2B) were regulated by EA treatment. In addition, protein expression of NR2B decreased in EA treatment. The NR2B subunit has been implicated in the modulation of learning, depression, and chronic pain [4, 34]. This finding is consistent with that reported by Wang et al. who showed that NMDAR dysregulation in the hippocampus is associated with increased anxiety-like behaviors and depressive symptoms [35]. Ryan et al. discovered distinct roles for the NR2A C-terminal domain (CTD) in regulating locomotor activity and impulsivity, whereas the NR2B CTD was involved in the regulation of perceptual learning, anxiety, impulsivity, and motor coordination [36, 37]. In addition, the suppression of NR2A or NR2B subunit-containing NMDARs in the hippocampus leads to the inhibition of anxiety-like behavior [38]. Collectively, these results suggest that EA treatment regulates NR2B receptor-mediated glutamate neurotransmission to reduce PTSD-like behavior. Furthermore, Fumagalli et al. demonstrated that acute stress does not impact the phosphorylation of NR1 at Ser 896 [39], whereas Calabrese et al. suggested that chronic stress increases the expression of the critical pNR1_(Ser896)_ subunit [40]. These findings suggest that the persistent elevation of glutamate levels in chronic stress or anxiety disorders could potentially lead to interactions with dysfunctional receptors at the postsynaptic level. In addition, activated NR1 results in the opening of ion channels, which may lead to the existence of a subunit in the endoplasmic reticulum and increased surface membrane localization [41]. Our results showed that the increased expression of pNR1(Ser896) was decreased following EA treatment. The data suggest that activation of the NR1 receptors may contribute to the upregulation of NR2B receptors through abnormal synaptic localization, even in the absence of direct evidence supporting this relationship. Moreover, our results showed that the number of GAD67-positive cells in the hippocampus of the SPSS model mice was attenuated by EA treatment. This is similar to the findings by Harvey et al., who demonstrated that GABA levels were decreased in the hippocampus of an SPS model [42]. These data suggest that EA treatment may regulate excitatory and inhibitory imbalances in the brain, which is an important aspect of the pathophysiology of PTSD.

Several studies have shown that neuronal immediate-early genes (IEGs) play important roles in synaptic processes by regulating synaptic strength, neuronal membrane properties, and neural circuit refinement. IEGs are rapidly and selectively upregulated in subsets of neurons in the hippocampus and other brain regions associated with learning, memory formation, and stress [43, 44]. Our results showed that the levels of the neuronal activity-related markers Arg 3.1 and Egr-1 were reduced by EA treatment. This supports the findings by Pacheco et al. who demonstrated increased mRNA levels of Arg 3.1 in the hippocampus of a chronic stress model [45]. IEGs are also associated with synaptic plasticity in memory recall [46, 47] and regulate it in an NMDA receptor-dependent manner [48]. Dendritic spines harbor glutamatergic synapses and mediate most excitatory synaptic transmission in the mammalian brain [49, 50]. Alterations in the morphology and number of dendritic spines are associated with synaptic development, maintenance, and plasticity [51]. Kwon et al. demonstrated that glutamate-induced spinogenesis requires opening of the NMDA receptor [52]. In our study, Golgi staining showed that the number of spines in the CA1 and DG regions of the hippocampus was decreased by EA treatment (Fig. 5). These results indicate that EA treatment modulates NR2B-associated excitatory synaptic neurotransmission to regulate neuronal activity in the PTSD model. Furthermore, NR2B plays a preferential role in the induction of synaptic plasticity, which is associated with fear extinction [53, 54]. However, our data suggests that EA treatment may block fear memory by regulating the NR2B receptor, although we did not investigate the stages of acquisition, consolidation, and retention of fear extinction. Further investigations are necessary to address this question more comprehensively.

Using magnetic resonance imaging, Bremner et al. demonstrated that hippocampal atrophy is implicated in PTSD [55, 56]. In addition, multiple studies have demonstrated that stress/glucocorticoid exposure induced glutamate excitotoxicity that culminated in neuronal damage and manifested as memory abnormalities and depression [21, 31, 35]. Moreover, NMDARs are primarily localized at the postsynaptic membrane and extrasynaptic receptors. Particularly, those containing the NR2B subunit play a significant role in mediating excitotoxicity. Our data showed that EA treatment regulated the expression of glutamate excitotoxicity-related mRNA (caspase-3 and Bax) and proteins (Bax, Bax/Bcl-2, and pJNK). Furthermore, caspase-3, a vital protein involved in apoptosis, is involved in the final stages of cellular damage [57]. Li et al. demonstrated that the expressions of Bax and caspase-3 and the Bax/Bcl-2 ratio increased by SPS in the hippocampal region [58, 59]. Collectively, our data suggest that EA effectively reduces glutamate excitotoxicity, potentially by preventing neuronal damage.

This study has some limitations. In our next study, we will investigate the localization of NR2B receptors, specifically examining whether they are predominantly found at synaptic or extra-synaptic sites, to determine the reason for glutamate excitotoxicity. Furthermore, we will conduct fear extinction studies to assess the effect of EA treatment on fear extinction. This investigation will help restore fear extinction early and prevent anxiety, depression, and cognitive impairment.

In conclusion, our results indicate the efficacy of EA treatment for PTSD-like behavior. EA treatment regulates the NR2B receptor to modulate glutamate transmission, leading to the attenuation of PTSD-like behavior. Collectively, the findings suggest that EA treatment at the GV29 acupoint is a promising therapeutic approach for PTSD. Moreover, EA treatment may be used as a complementary modality to drug treatment with SSRI, increasing the therapeutic effect for PTSD patients through regulation of more neurotransmitters.

## Data Availability

The data that support the findings of this study are available by reasonable request.
